# In Silico Detection of Antimicrobial Resistance Integrons in *Salmonella enterica* Isolates from Countries of the Andean Community

**DOI:** 10.3390/antibiotics10111388

**Published:** 2021-11-12

**Authors:** Lilibeth Torres-Elizalde, David Ortega-Paredes, Karen Loaiza, Esteban Fernández-Moreira, Marco Larrea-Álvarez

**Affiliations:** 1 School of Biological Science and Engineering, Yachay-Tech University, Hacienda San José, Urcuquí 100650, Ecuador; lilibeth.torres@yachaytech.edu.ec; 2 Unidad de Investigación de Enfermedades Transmitidas por Alimentos y Resistencia a los Antimicrobianos (UNIETAR), Facultad de Medicina Veterinaria y Zootecnia, Universidad Central del Ecuador, Quito 170129, Ecuador; daortegap@uce.edu.ec; 3 Facultad de Ciencias Médicas Enrique Ortega Moreira, Carrera de Medicina, Universidad Espíritu Santo, Km 2.5 vía Samborondón, Samborondón 0901952, Ecuador; 4 Research Unit, Life Science Initiative (LSI), Quito 170102, Ecuador; karenloaizaconza@gmail.com

**Keywords:** *Salmonella enterica*, class 1 and 2 integrons, *dfrA* genes, bioinformatic tools, IntFinder, Andean Community

## Abstract

Antimicrobial resistance genes are often associated with integrons, which promote their movement between and within DNA molecules. IntFinder 1.0 and I-VIP v1.2 were used for the detection of integrons and their associated resistance genes in assembled sequences and raw reads. A dataset comprising 1688 sequenced *Salmonella enterica* isolates from countries of the Andean Community was developed. A total of 749 and 680 integrons were identified by IntFinder 1.0 and I-VIP v1.2, respectively; class 2 integrons were the most abundant followed by class 1, whereas no class 3 integrons were detected. These elements were mainly associated with isolates from animal sources. *S.* Infantis ST32 contained the majority of integrons. Trimethoprim resistance genes (*dfrA*) were found in greater numbers than others, including *aadA* and *bla* genes. The presence of these resistance integrons may come as a response to antibiotic misuse, especially of co-trimoxazole. This represents a public health risk as novel resistant strains might appear due to gene dissemination. The information gathered from in silico studies not only contributes to our understanding of integron dynamics in pathogenic *Salmonella*, but also helps identify potential emergent patterns of resistance in the region, which is fundamental for developing pertinent antibiotic surveillance programs.

## 1. Introduction

*Salmonella enterica* is a Gram-negative bacterium that can infect humans and animals; it is mostly transmitted to humans in contaminated foods and represents one of the leading causes of bacterial foodborne diseases. The emergence of multidrug-resistant (MDR) strains can affect antibiotic treatments and may increase mortality rates of *S. enterica* infections [1]. Strains of *S. enterica* resistant to commonly used antibiotics have been reported in country members of the intergovernmental organization known as the Andean Community, composed of Colombia, Ecuador, Peru, and Bolivia [2,3,4,5,6].

Antibiotic resistance traits can arise from random mutations in the genome or can be acquired through horizontal gene transfer [7,8]. Pre-existing resistance determinants can be collected from the gene pool by the combined activity of mobile genetic elements capable of moving between and within DNA molecules, such as gene cassettes/integrons, insertion sequences and transposons, and those able to transport between bacterial cells, which include integrative conjugative elements and plasmids [9]. Integrons, in particular, are associated with conjugative plasmids that permit their mobilization between different bacterial cells [10,11].

Integrons are genetic platforms able to capture, transport, and integrate gene cassettes into specific locations from which gene expression can take place [12]. Gene cassettes are free circular DNA sequences that contain an open reading frame, occasionally with its own promoter, and a recombination site (*attC*). Within the integron sequence, an integrase is encoded by the *intI* gene that recognizes the *attC* site and catalyzes the insertion of the cassette into the integron at the recombination site known as *attI.* The *intl* gene, its promoter, and the *attI* site, containing a promoter for the inserted cassette, constitute the 5′ conserved sequence (5′ CS) of the integron [13,14]. Integrons are grouped in classes based on the integrases encoded by three different genes: *intI1*, *intI2*, and *intI3.* Class 1 integrons are associated with *Intl1*, while integrons class 2 and 3 are associated with *Intl2* and *Intl3*, respectively. The 3′ CS might contain several open reading frames, depending on the integron class, conferring resistance to antibiotics (sulfonamides) and bactericidal compounds (e.g., quaternary ammonium). Class 1 integrons normally carry such genes, whereas class 2 integrons contain genes encoding for transposition proteins (*tns*). No sequences have been reported in the 3′ CS of class 3 integrons [15]. 

Resistance genes can be inserted as cassettes into integrons and then mobilized to other regions of the genome, including conjugative elements. This phenomenon results in the appearance of a variety of multidrug resistance patterns in bacteria [16,17]. Undoubtedly, the interaction of these elements forms a complex system with the capacity of recruiting and spreading resistance genes in bacterial populations. Thus, the identification and characterization of integrons seem crucial for assessing the epidemiology of antibiotic resistance genes, especially in areas where information is scarce. Class 1 and 2 integrons have been detected in multidrug-resistant isolates of *Salmonella enterica* reported in Peru and Colombia [18,19,20], while no reports were found from Ecuador and Bolivia. The lack of information regarding integrons limits the capacity to study the dynamics of these elements and their association with antibiotic resistant genes.

Genomic analyses are more readily available today due to the rapid development of next-generation sequencing. Traditional bioinformatic tools used for detecting mobile genetic elements require moderate training for operation, and most are focused on elements such as insertion sequences, integrative mobilizable elements, and plasmids. The development of accessible software has proved advantageous for investigating mobile elements in pathogenic bacteria [21,22]. Here, we utilized IntFinder 1.0 (Center for Genomic Epidemiology—Technical University of Denmark, Kongens. Lyngby, Denmark), a program that allows detection of integrons 1, 2, and 3, using raw reads and assembled genomes/contigs as input [23], as well as I-VIP v1.2 (University of Hong Kong, Pok Fu Lam, Hong Kong), a pipeline developed to detect integrons in complete genomes [24], to identify these elements and studying their relationship with antibiotic resistance genes in isolates reported in countries of the Andean Community.

## 2. Results

### 2.1. Final Dataset

The developed dataset contained 1688 isolates of *S*. enterica, for which FASTA files were downloaded in February 2021. Of these isolates, 889 (52.67%) were from Ecuador, 466 (27.61%) were from Colombia, 274 (16.23%) were from Peru, 50 (2.96%) were from Venezuela, and nine (0.53%) were from Bolivia (Table S1). The majority of isolates (46.98%) were associated with animal samples, while 25.95% were related to human sources; 16.82% of isolates came from to food products and 9.95% came from the environment. Five isolates (0.30%) were classified as undetermined. 

### 2.2. Characterization of Detected Integrons

**Figure 1:**
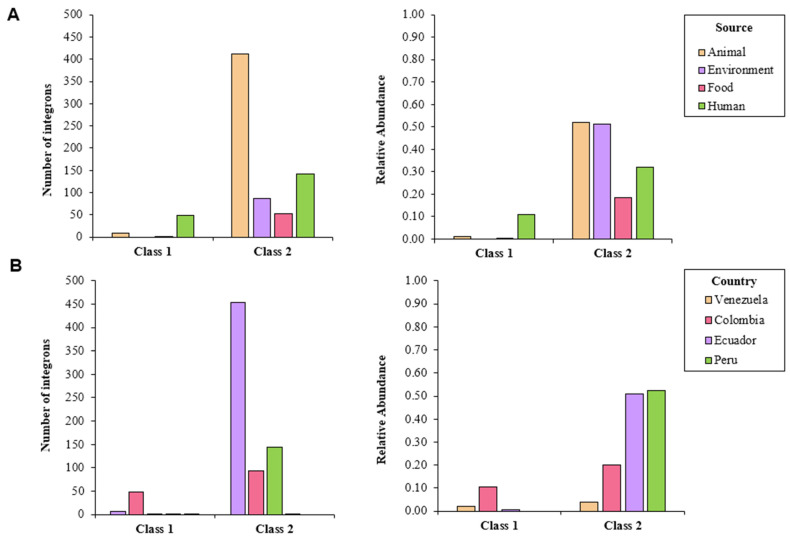
Number of detected integrons in whole-genome sequenced isolates of *S. enterica* according to IntFinder 1.0, classified by (**A**) source and (**B**) country.

Overall, 749 integrons were predicted by IntFinder 1.0, with the majority of them being class 2 (92.4%), followed by class 1 integrons (7.6%); no class 3 integrons were detected (Table S2). These elements were predicted in isolates from all sources. Half of the isolates from environmental and animal samples contained class 2 integrons. Isolates from human samples contained mostly class 1 integrons; genomes from animal sources also carried some of these elements (Figure 1A). Class 1 and 2 integrons were detected in isolates from all the countries studied, with the exception of Bolivia. More than 50% of the isolates coming from Peru and Ecuador contained class 2 integrons, whereas a lower abundance was found in Colombia and Venezuela. Class 1 integrons were detected in samples documented in Colombia, as well as in those from Venezuela and Ecuador (Figure 1B).

**Figure 2:**
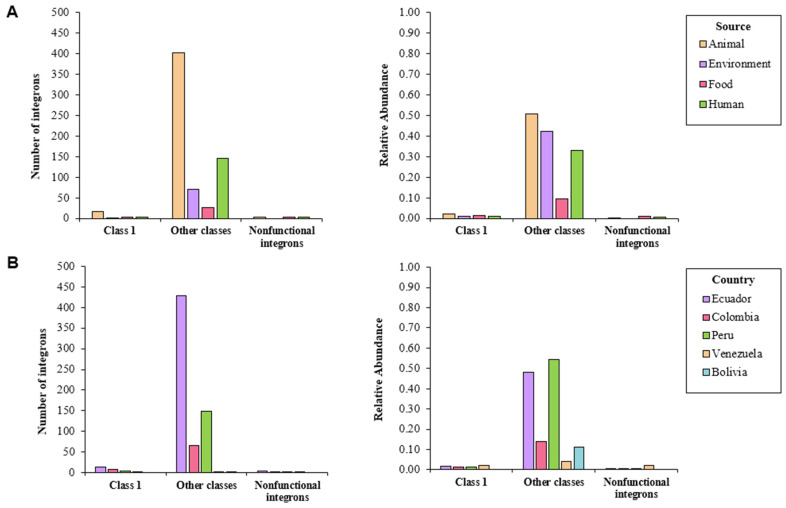
Number of integrons detected in whole-genome sequenced isolates of *S. enterica* according to I-VIP v1.2, classified by (**A**) source and (**B**) country.

I-VIP v1.2 detected a total of 680 integrons; those classified as other classes were the most common (94.9%), followed by class 1 (3.8%) and nonfunctional integrons (1.3%) (Table S2). More than 40% of the isolates coming from animal and environmental sources contained integrons categorized as other classes; only 33% of human samples and 10% of food samples harbored these elements. The latter two sources were proven to carry nonfunctional integrons (1%), while class 1 integrons were detected in less than 2% of the isolates from all sources (Figure 2A). Around half of the isolates reported in Peru and Ecuador harbored the integrons categorized as other classes; less than 15% of the screened genomes coming from Colombia, Bolivia, and Venezuela contained these elements. Nonfunctional genomes were found in isolates from Colombia and Venezuela (Figure 2B).

### 2.3. Differences between MLST Types and Serovars

**Figure 3:**
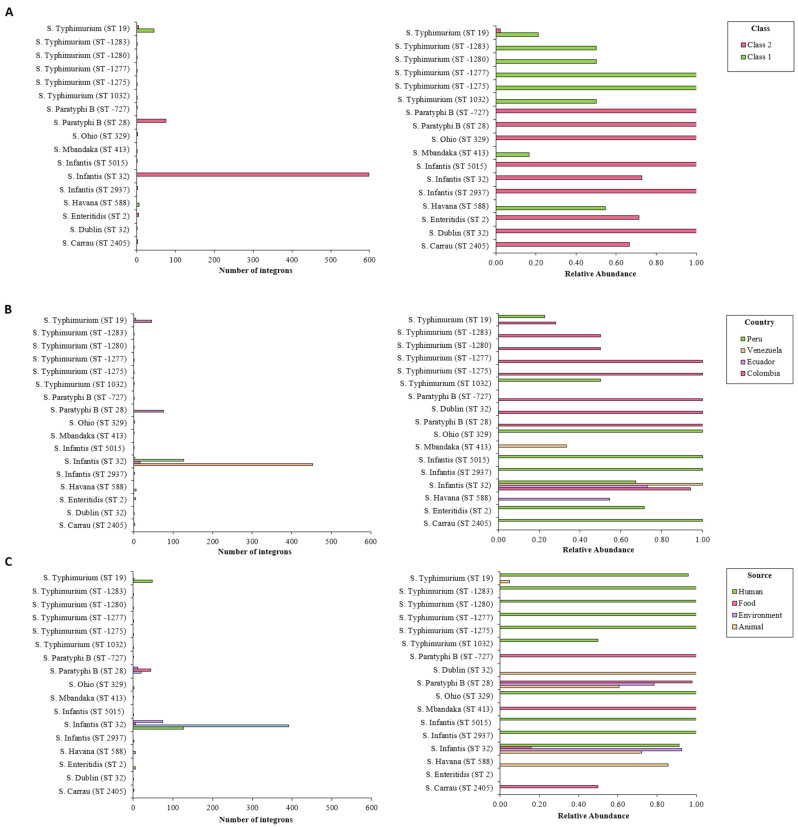
Number of integrons detected by IntFinder 1.0 according to sequence type, organized by (**A**) integron class, (**B**) source, and (**C**) country. *Int*, integrase.

The integrons predicted by IntFinder 1.0 varied according to the sequence type and serovar. The most abundant serovars (*S*. Infantis, *S*. Paratyphi, and *S*. Typhimurium) proved to contain most of these elements. More than 70% of the *S.* Infantis ST32 isolates harbored integrons, all of them class 2. All isolates from other sequence types belonging to this serovar were positive for class 2 integrons. These elements proved to be present in all the *S.* Paratyphi ST28 genomes analyzed. Around 20% of *S.* Typhimurium ST19 isolates carried integrons. Other sequence types of this serovar, despite being less common, showed higher relative abundance of these elements. Genomes from other serovars, not present in large numbers, had nonetheless more integrons per isolate (Figure 3A). *S*. Infantis ST32, *S*. Paratyphi ST28, and *S*. Typhimurium ST19 contained most of the predicted integrons. These isolates were associated with all sources and countries, excepting Bolivia. Less common isolates were linked to various sources from several countries (Figure 3B,C).

**Figure 4:**
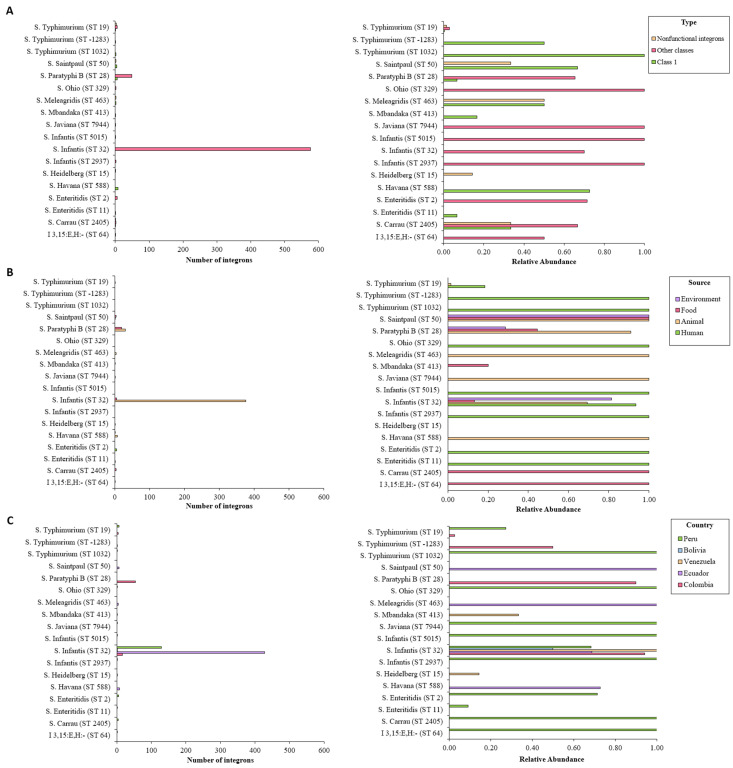
Number of integrons detected by I-VIP v1.2 according to sequence type, organized by (**A**) integron class, (**B**) source, and (**C**) country. *Int*, integrase.

According to I-VIP v1.2, both *S*. Infantis ST32 and *S*. Paratyphi ST28 contained most of the detected integrons, which corroborated the results obtained with IntFinder 1.0. More than 70% of the genomes belonging these serovar/STs proved to be integron positive. On the other hand, only 4% of *S*. Typhimurium ST19 isolates contained these elements. As shown previously, less common isolates proved to have high relative abundance of integrons (Figure 4A). Common serovars ST were associated with integrons from other classes coming from all studied countries (Figure 4B,C).

**Figure 5:**
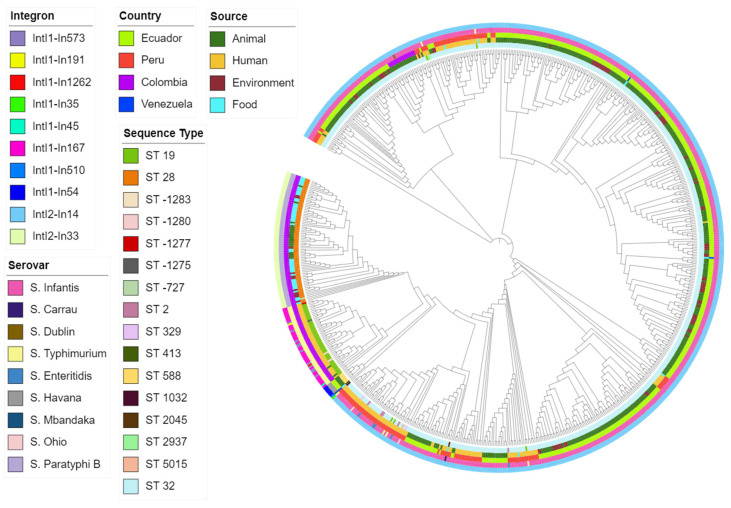
Maximum-likelihood phylogenetic tree of all isolates based on integron profile. Covering predicted integron, source, country, serovar, and sequence type.

Isolates from the same serovar ST carried similar integrons. Some integrons were found in specific backgrounds, while others were associated with various sources and countries. As the outcomes from the two programs were similar, we used the IntFinder 1.0 data to develop the phylogenetic tree (Figure 5).

### 2.4. Association of Integrons and Antibiotic Resistance Genes

**Figure 6:**
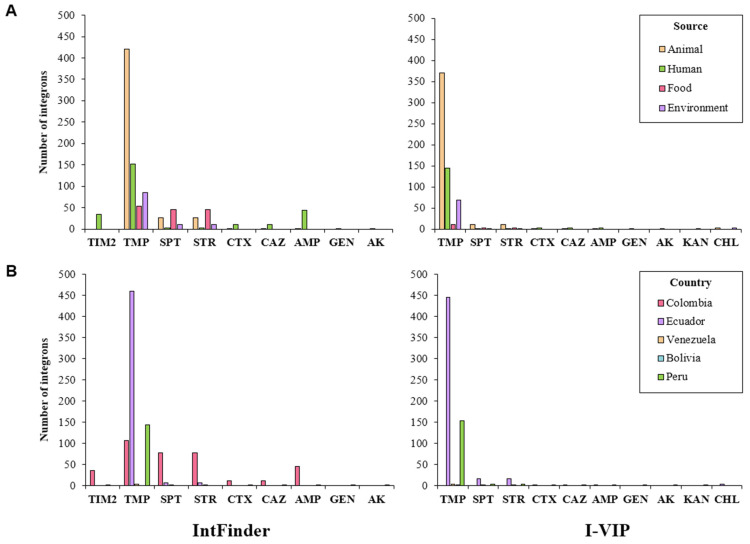
Number of integrons associated with antimicrobial resistance genes, organized by (**A**) source and (**B**) country. SPT, spectinomycin; STR, streptomycin; TMP, trimethoprim; AMP, ampicillin; CTX, cefotaxime; CAZ, ceftazidime; TIM2, ticarcillin/clavulanic acid; GEN, gentamicin; AK; akamicin; KAN; kanamycin.

Trimethoprim resistance genes were the most abundant. These genes were linked to integrons predicted in isolates from all sources, documented mainly in Ecuador, Colombia, and Peru. Genes conferring resistance to spectinomycin/streptomycin were detected in isolates from different sources reported principally in Colombia and to a lesser extent in Ecuador. Resistance genes related to other antimicrobials (ampicillin, cefotaxime, ceftazidime, gentamicin, akamicin, kanamycin, chloramphenicol, and ticarcillin/clavulanic acid) were also predicted by the two programs (Figure 6). 

According to IntFinder 1.0, eight different gene cassettes were detected among the identified integrons. Genes encoding for dihydrofolate reductases (*dfr*), which bestow resistance to trimethoprim, were present in most of the cassettes. Genes conferring resistance to aminoglycosides, β-lactams, and cephalosporins were also identified (Table 1).

I-VIP v1.2 revealed the presence of nine different cassettes among the predicted integrons. Again, *dfr* genes were the most abundant, although genes encoding for acetyl-, adenyl-, and phosphotransferases, as well as those encoding for β-lactamases, were also detected (Table 2).

## 3. Discussion

Studies regarding the identification and characterization of integrons in *Salmonella enterica* in this region are scarce. In Peru and Colombia, class 1 and 2 integrons have been detected in multidrug-resistant isolates of *Salmonella enterica* [18,19,20]. No reports were found from Ecuador and Bolivia. Information regarding integrons in countries of the Andean Community seems insufficient to draw conclusions of their influence in the emergence of resistant strains. Previous research has demonstrated that the application of bioinformatic tools is propitious for identifying mobile elements and determining their relationship with antimicrobial resistance genes in zoonotic bacteria [21,22]. IntFinder 1.0 is a program that permits integron detection using both assembled genomes/contigs and raw reads as input [23]. I-VIP v1.2 is a pipeline that allows detection of class 1 integrons in assembled metagenomes and complete/draft genomes [24]. These software could be considered advantageous when compared to previous tools that only accept assembled genomes/contigs [24,25,26]. 

The dataset developed revealed that more isolates were reported from Ecuador (52.67%) than from other countries, which resulted in Ecuador being the country with the highest number of integrons. However, integron relative abundance was similar for Ecuador, and Peru, Colombia, Venezuela, and Bolivia showed lower abundance of these elements. A marked difference was observed in the number of integrons carried by strains of *S. enterica* associated with different serovar/sequence types. As shown here, *S.* Infantis and *S.* Paratyphi B have been associated with both class 1 and 2 integrons [27,28]. There are some reports documenting the presence of class 1 and 2 integrons in *S.* Typhimurium [17,29,30]. The other detected serovars have been indeed related to these elements.

The predicted integrons were linked to genes that not only confer resistance to antimicrobial agents used as the traditional first line of defense against salmonellosis, such as trimethoprim or ampicillin, but also to third-generation cephalosporins and to different aminoglycosides [31]. The use of these antibiotics is widespread in the region, for both animal production and human health [32,33,34,35]. Unsurprisingly, isolates from animal sources proved to be linked with the vast majority of resistance genes, followed by isolates from human, food, and environmental sources. Despite the number of detected integrons, only a limited number of coding sequences were identified within the variable regions. The most abundant integron was linked to *dfrA14*. This gene encodes for a type A TMP-resistant dihydrofolate reductase; *dfrA*s are expressed along with TMP-sensitive *folA*, which permits folate to be produced and thus bacterial survival [36]. *S.* Infantis ST32 was linked to *dfrA14*. Indeed, in Ecuador and Colombia, strains of *S.* Infantis resistant to TMP/SUL have been reported [2,37]. This resistance has been associated with *drfA*s genes, although no links to class 2 integrons were assessed [37]. In Peru, strains from this serovar proved to carry *dfrA*s genes, but no relationship with integrons were detected [4]. Nevertheless, the association of these genes with class 2 integrons in isolates from countries of other regions has been previously detected [38,39]. The other class 2 integron was associated with a different DfrA, encoded by *dfrA1*. This cassette was accompanied by another containing a gene encoding an aminoglycoside adenylyltransferase (*aadA1*) that *O*-adenylates position 3″ of streptomycin and position 9 of spectinomycin [40]. The *dfrA1* and *aadA1* genes have been related to strains of *S.* Paratyphi documented in Colombia, no associations were described with class 2 integrons [3,5]. These genes, however, have been linked to class 1 integrons in isolates from *S. enterica* [41,42]. A similar cassette has been reported in other pathogenic bacteria; this cassette has been associated with class 2 integrons [38,43,44]. 

Class 1 integrons were associated with different resistance patterns as their cassettes contained genes encoding for Dfrs, AadAs, Aphs, CmlAs, and Aacs. Serovars containing the aforementioned genes have been linked to *S. enterica* serovars such as *S.* Typhimurium and *S.* Enteritidis [45,46]. The gene cassette associated with class 1 integrons also carried the *blaOXA-2* genes. The OXA-type β-lactamases (OXA-15, OXA-2) are known to confer resistance to ampicillin, cefotaxime, and ceftazidime [47]. Lastly, *blaCARB2* genes were also detected, which encode for a class A β-lactamase known for providing resistance to ampicillin and ticarcillin [48]. *S.* Typhimurium ST19 proved to be associated with DfrA29 and the mentioned β-lactamases. Actually, strains of *S.* Typhimurium resistant to trimethoprim and ampicillin have been documented in Colombia and linked to the presence of *dfr*s, *blaCARB2*, and *blaOXA-2* genes, although no association with integrons was assessed [3].

Sulfonamides combined with trimethoprim are widely applied to food-producing animals [49,50]. The latter is presented under the name of co-trimoxazole (trimethoprim/sulfamethoxazole; STX), which is of common use in the studied area [51,52,53]. In fact, several studies have reported resistant strains of *S.* Infantis, mainly derived from poultry production, in countries of the Andean Community [4,37,54]. Such resistance proved to be associated with the presence of *dfr* genes [3,4], as reported in countries of other regions [27,39,55]. The extensive use of the drug might present a selective pressure that favors integrons associated with dihydrofolate reductases. In serovars associated with class 1 integrons, resistance to STX could be linked to these elements as they bear *dfr* genes. These integrons commonly contain genes (*sul*) providing resistance to sulfonamides in their 3′ CS [15]. On the other hand, serovars linked to class 2 integrons are not associated with *sul* genes. Nevertheless, strains of *S.* Infantis reported in Ecuador and Peru have been linked to *sul* genes [4,37]. Likewise, strains of *S.* Paratyphi B have been related to these sequences [56]. In these studies, the association between integrons and these genes was not assessed. Undoubtedly, resistance integrons might be playing a key role in the development of resistant strains to the bactericidal drug co-trimoxazole. Furthermore, these integrons were related to other genes providing resistance to commonly used antibiotics, including aminoglycosides and β-lactams [49,50]. Integrons could be incorporated into structures such as plasmids and integrative elements, whereby their associated resistance genes could be spread among bacterial populations [48,57,58]. Indeed, the *dfrA14* and *aadA1* genes have been linked to pESI-like plasmids reported in *S.* Infantis isolated from broilers and chicken food products [59,60]. pESI-like plasmids carrying *aadA1*-associated integrons have been reported in Peru [61]; such plasmids have also been documented in Ecuador, although no relationship with integrons was assessed [37].

Evidently, strains of *S. enterica* resistant to trimethoprim and to other commonly used antibiotics are prevalent in countries of the Andean Community. However, such resistance has not been extensively associated with integrons and their cassettes. The present outcomes suggest that the detected integrons might be playing an important role in the appearance of resistant strains. Arguably, therefore, the observed prevalence of *dfrA*s-associated integrons, coming principally from broiler samples, should be considered of high risk as these genes are associated with complex mobile structures that facilitate their dissemination in the environment.

## 4. Materials and Methods

### 4.1. Selection of Dataset

Whole-genome sequenced isolates of *S. enterica* were retrieved from the EnteroBase (http://enterobase.warwick.ac.uk/) (accessed on 30 November 2020), an online platform devised for analyzing genomic variation within enteric bacteria. For this research, a *Salmonella* spp. dataset was created on the basis of the following criteria: (i) collected in countries of the Andean Community (Colombia, Ecuador, Peru, and Bolivia; Venezuela was included as it was an official member until 2006), (ii) reported between 1956 and 2021, and (iii) nonrepetitive whole genomes. FASTA files of the isolates were downloaded in February 2021. In order to identify resistance integrons in the developed database, two approaches were investigated. The first used the IntFinder 1.0 (Center for Genomic Epidemiology—Technical University of Denmark, Kongens. Lyngby, Denmark) (software version: 2019-12-18; database version: 2019-11-29), a program targeting class 1, 2, and 3 integrons in assembled genomes/contigs and raw reads as input [23]. The second approach replicated the methodology used in a previous study [24], which consists of a pipeline to identify class 1 integrons in complete/draft genomes and assembled metagenomes. Both schemes aimed at detecting integrons and determining their association with antibiotic resistance genes.

### 4.2. IntFinder 1.0

IntFinder 1.0 was developed with Python3 [23]. It is accessible from the following web server: https://cge.cbs.dtu.dk/services/IntFinder-1.0/ (accessed on 18 January 2021) IntFinder 1.0 was originally developed to detect class 1 integrons, although the version used herein is also able to detect class 2 and 3 integrons. Databases were developed using a publicly available repository, INTEGRALL (http://integrall.bio.ua.pt/) (accessed on 18 January 2021). This web-based platform compiles crucial information about integrons, including their sequence, genetic context, and molecular arrangement [25].

All databases were constructed on the basis of the number assigned to each integron (*In*) by INTEGRALL. Each *In* contains information regarding gene cassettes, integron names, and GenBank accession numbers. First, all nucleotide sequences were collected from NCBI’s gene database using the GenBank accession numbers. Then, sequences were blasted to detect the 5′CS and 3′CS ends of integrons, as well as their associated resistance genes. Integron nucleotide coordinates were determined using ResFinder (Center for Genomic Epidemiology—Technical University of Denmark, Kongens. Lyngby, Denmark) (software version: 2019-01-29; database version: 2019-02-20) [62].

IntFinder 1.0 carried out identifications through KMA 1.3.9. (Center for Genomic Epidemiology—Technical University of Denmark, Kongens. Lyngby, Denmark)) [63] with k-mer alignment of the whole-genome sequenced isolate under investigation to the entry sequences of the database according to a user-defined similarity threshold. In the web server version, the minimum threshold is 0.5, whereas, in the software version, this can be adjusted as desired. Integron classes were manually identified according to the sequence of the *intI* gene. Potential class 1 integrons were marked as such if the *intI1* sequence was detected on the contig. If, on the other hand, *intI2* was detected therein, integrons were marked as class 2. The sequence of *intI3* was used to detect class 3 integrons. Elements carrying similar integrases were considered members of the same class. Class 1–3 integrons were predicted using IntFinder 1.0, according to the method described in Section 2.1. An integron was considered present only if it matched correctly to the reference sequences (coverage = 1, sequence identity = 1, depth = 1). Resistance genes were classified as being associated with different integrons. Genes were considered associated if located within the integron variable region.

As a result, IntFinder 1.0 produced four files for each query as result: (i) results.txt, which contained information regarding name, query/template length, resistance genes and positions, and accession numbers; (ii) results.tsv, this file contained the graphical alignment between the input sequence and the integron entry of the database, along with information about length and associated genes; (iii) Hit_in_genome_seq.fsa, which contained information about overage, identity, and match; (iv) Integrons.fsa, which included the sequence of the detected integron.

The phylogenetic tree was rendered with CSI Phylogeny 1.4 (https://cge.cbs.dtu.dk/services/CSIPhylogeny/, accessed on 3 November 2021); the visualization and annotation were carried out using iTOL v6 (https://itol.embl.de/, accessed on 4 November 2021). 

### 4.3. I-VIP v1.2

The Integron Visualization and Identification Pipeline (I-VIP v1.2) was used for identifying, classifying, annotating, and visualizing class 1 integrons in the aforementioned isolates. The analysis was carried out as described previously [24]. Three elements were used for detection: the sequence of the *intI1* gene (5′ CS), the *attC* site linked to gene cassettes, and the sulfonamide resistance gene (*sulI*) (3′ CS). 

Identification was carried out against reference databases: the *attC* database [26] (e-value of 1 for 2 Mb) by cmsearch 1.1.1 (Eddy/Rivas Laboratory—Harvard University, Cambridge, MA, USA) [64]; the integrase database [24] (e-value of 1 × 10^−3^, 80% aa similarity over 50% aa hit length); the *sulI* database [65] (e-value of 1 × 10^−3^, 90% aa similarity over 80% aa hit length) by BLASTP 2.2.28+ (National Center for Biotechnology Information, Bethesda, MD, USA) [66]. Gene cassettes were extracted and annotated using the SARG [67] (e-value of 1 × 10^−5^, 90% aa similarity over 80% aa hit length) and ComMet [67] (e-value of 1 × 10^−5^, 80% aa similarity over 90% aa hit length) databases by BLASTP 2.2.28+ [66]. 

According to the integron elements, I-VIP v1.2 classified the predicted integrons into five types (A–E), with type A being those carrying both *intI1* and *sulI* genes, whereas those containing only the *intI1* sequenced are referred to as type B. Integrons harboring integrases other than class 1 are called type C. Type D integrons contain *sulI* genes but no integrases. Type E integrons contain neither *sulI* genes nor an integrase; the latter two types are classified as nonfunctional integrons [24]. For this research, we classified integrons intro three classes: (i) class 1 integrons, which comprised those classified as type A or type B; (ii) other classes, which involved type C integrons; (iii) nonfunctional integrons, including types D and E. 

## 5. Conclusions

IntFinder 1.0 and I-VIP v1.2 proved to be valuable tools for characterizing integrons and their relationship to antibiotic resistance genes in *S. enterica* isolates reported in countries of the Andean community. Class 2 integrons were the most common; these elements were primarily dependent on serovar ST rather than isolate source and country of origin. Additionally, trimethoprim resistance genes (*dfr*) were highly prevalent among the cassettes detected in integrons class 1 and 2; these cassettes also carried resistance genes for aminoglycosides and β-lactams. The presence of these resistance genes might be a response to antibiotic misuse, particularly co-trimoxazole. Integrons are known to be carried on plasmids and other integrative elements, whereby the associated resistance genes can be disseminated between bacterial species; this represents a public health risk as novel resistant strains might appear. The information gathered from in silico studies is useful not only for understanding an important part of the mobilome in pathogenic *Salmonella*, but also for recognizing emergent patterns of resistance in the region and, thus, assisting in designing appropriate surveillance programs. 

## Figures and Tables

**Table 1:** Antimicrobial resistance genes detected among integrons identified in whole-genome sequenced isolates of *Salmonella enterica* from countries of the Andean Community according to IntFinder 1.0.

Integrase	Gene Cassettes in the Variable Region	Associated Resistance Pattern	Name	Length (bp)	Accession Number
*IntI1*	*dfrA17*, *aadA5*	TMP–SPT–STR	*In54*	474–789	AF220757
*IntI1*	*blaCARB-2*	AMP–TIM2	*In167*	866	AF221899
*IntI1*	*dfrA29*, *blaOXA-2*	TMP–AMP–CTX–CAZ	*In573*	472–828	AM237806
*IntI1*	*dfrA12-aadA2*	TMP–SPT–STR	*In1262*	497–791	KX710093
*IntI1*	*dfrA14*	TMP	*In191*	473	HF545433
*IntI1*	*aac(6’)-Ib-cr-aac(6’)-Ib3-blaOXA-2-blaOXA-34*	GEN–AK–AMP–CTX–CAZ	*In35*	587–554–827–770	DQ139276
*IntI1*	*aadA12*	TMP	*In45*	791	FJ460240
*IntI1*	*dfrA12-aadA2*	TMP–SPT–STR	*In510*	498–792	EU853659
*IntI2*	*dfrA14*	TMP	*In14*	483	EU780012
*IntI2*	*dfrA1*, *aadA1*	TMP–SPT–STR	*In33*	475	FJ914220

SPT, spectinomycin; STR, streptomycin; TMP, trimethoprim; AMP, ampicillin; CTX, cefotaxime; CAZ, ceftazidime; TIM2, ticarcillin/clavulanic acid; GEN, gentamicin; AK, akamicin.

**Table 2:** Antimicrobial resistance genes detected among integrons identified in whole-genome sequenced isolates of *Salmonella enterica* from countries of the Andean Community according to I-VIP v1.2.

Integrase	Gene Cassettes in the Variable Region	Associated Resistance Pattern	Type
*IntI1*	*aac(6)-I-OXA-2*	GEN–AK–AMP–CTX–CAZ	Class 1
*IntI1*	*OXA-2*	AMP–CTX–CAZ	Class 1
*IntI1*	*aph(3)-I-aph(6)-I-dfrA1-aadA*	KAN–TMP–SPT–STR	Class 1
*IntI1*	*dfrA12-aadA*	TMP–SPT–STR	Class 1
*IntI1*	*dfrA17-aadA*	TMP–SPT–STR	Class 1
*IntI1*	*dfrA12-aadA-cmlA-aadA*	TMP–SPT–STR–CHL	Class 1
*IntI1*	*aadA*	SPT–STR	Class 1
Other	*dfrA14*	TMP	Other integrases
Other	*dfrA14-dfrA14*	TMP	Other integrases
No integrase	*aadA*	SPT–STR	Nonfunctional
No integrase	*dfrA1-aadA*	TMP–SPT–STR	Nonfunctional

SPT, spectinomycin; STR, streptomycin; TMP, trimethoprim; AMP, ampicillin; CTX, cefotaxime; CAZ, ceftazidime; TIM2, ticarcillin/clavulanic acid; GEN, gentamicin; AK; akamicin; KAN; kanamycin.

## Data Availability

Data are available on request.

## Supplementary Materials

The following are available online at https://www.mdpi.com/article/10.3390/antibiotics10111388/s1. Table S1. Dataset of whole-genome-sequenced *S. enterica* isolates in the Andean Region; Table S2. Distribution of resistance genes among detected resistance integrons in *Salmonella enterica* isolates in the Andean Region.

## References

[1] Parisi A., Crump J.A., Glass K., Howden B.P., Furuya-Kanamori L., Vilkins S., Gray D.J., Kirk M.D. (2018). Health Outcomes from Multidrug-Resistant *Salmonella* Infections in High-Income Countries: A Systematic Review and Meta-Analysis. Foodborne Pathog. Dis..

[2] Vinueza-Burgos C., Cevallos M., Ron-Garrido L., Bertrand S., De Zutter L. (2016). Prevalence and Diversity of Salmonella Serotypes in Ecuadorian Broilers at Slaughter age. PLoS ONE.

[3] Ramirez-Hernandez A., Carrascal-Camacho A.K., Varón-García A., Brashears M.M., Sanchez-Plata M.X. (2021). Genotypic characterization of antimicrobial resistant *Salmonella* spp. Strains from three poultry processing plants in Colombia. Foods.

[4] Vallejos-Sánchez K., Tataje-Lavanda L., Villanueva-Pérez D., Bendezú J., Montalván Á., Zimic-Peralta M., Fernández-Sánchez M., Fernández-Díaz M. (2019). Whole-Genome Sequencing of a Salmonella enterica subsp. enterica Serovar Infantis Strain Isolated from Broiler Chicken in Peru. Microbiol. Resour. Announc..

[5] Donado-Godoy P., Bernal J.F., Rodríguez F., Gomez Y., Agarwala R., Landsman D., Mariño-Ramírez L. (2015). Genome sequences of multidrug-resistant *Salmonella enterica* serovar Paratyphi B (dT+) and Heidelberg strains from the Colombian poultry chain. Genome Announc..

[6] Fernandez J., Fica A., Ebensperger G., Calfullan H., Prat S., Fernandez A., Alexandre M., Heitmann I. (2003). Analysis of molecular epidemiology of Chilean *Salmonella enterica* serotype enteritidis isolates by pulsed-field gel electrophoresis and bacteriophage typing. J. Clin. Microbiol..

[7] Zankari E., Allesøe R., Joensen K.G., Cavaco L.M., Lund O., Aarestrup F.M. (2017). PointFinder: A novel web tool for WGS-based detection of antimicrobial resistance associated with chromosomal point mutations in bacterial pathogens. J. Antimicrob. Chemother..

[8] Huddleston J.R. (2014). Horizontal gene transfer in the human gastrointestinal tract: Potential spread of antibiotic resistance genes. Infect. Drug Resist..

[9] Partridge S.R., Kwong S.M., Firth N., Jensen S.O. (2018). Mobile genetic elements associated with antimicrobial resistance. Clin. Microbiol. Rev..

[10] Che Y., Yang Y., Xu X., Rinda K.B., Polz M.F., Hanage W.P., Zhang T. (2021). Conjugative plasmids interact with insertion sequences to shape the horizontal transfer of antimicrobial resistance genes. Proc. Natl. Acad. Sci. USA.

[11] Martínez N., Mendoza C.M., Rodríguez I., Soto S., Bances M., Rodicio M.R. (2007). Detailed structure of integrons and transposons carried by large conjugative plasmids responsible for multidrug resistance in diverse genomic types of *Salmonella enterica* serovar Brandenburg. J. Antimicrob. Chemother..

[12] Escudero J.A., Loot C., Mazel D. (2018). Integrons as Adaptive Devices. Grand Challenges in Biology and Biotechnology.

[13] Michael C.A., Labbate M. (2010). Gene cassette transcription in a large integron-associated array. BMC Genet..

[14] Jacquier H., Zaoui C., Sanson-Le Pors M.J., Mazel D., Berçot B. (2009). Translation regulation of integrons gene cassette expression by the attC sites. Mol. Microbiol..

[15] Deng Y., Bao X., Ji L., Chen L., Liu J., Miao J., Chen D., Bian H., Li Y., Yu G. (2015). Resistance integrons: Class 1, 2 and 3 integrons. Ann. Clin. Microbiol. Antimicrob..

[16] Doublet B., Zhao H., Chen W., Xu X., Zhou X., Shi C. (2018). Transmissible ST3-IncHI2 Plasmids Are Predominant Carriers of Diverse Complex IS26-Class 1 Integron Arrangements in Multidrug-Resistant *Salmonella*. Front. Microbiol..

[17] Argüello H., Guerra B., Rodríguez I., Rubio P., Carvajal A. (2018). Characterization of Antimicrobial Resistance Determinants and Class 1 and Class 2 Integrons in *Salmonella enterica* spp., Multidrug-Resistant Isolates from Pigs. Genes.

[18] O’mahony R., Quinn T., Drudy D., Walsh C., Whyte P., Mattar S., Fanning S. (2006). Antimicrobial Resistance in Nontyphoidal *Salmonella* from Food Sources in Colombia: Evidence for an Unusual Plasmid-Localized Class 1 Integron in Serotypes Typhimurium and Anatum. Microb. DRUG Resist..

[19] Martínez-Puchol S., Riveros M., Ruidias K., Granda A., Ruiz-Roldán L., Zapata-Cachay C., Ochoa T.J., Pons M.J., Ruiz J. (2021). Dissemination of a multidrug resistant CTX-M-65 producer *Salmonella enterica* serovar Infantis clone between marketed chicken meat and children. Int. J. Food Microbiol..

[20] Ricardo Castellanos L., van der Graaf-Van Bloois L., Donado-Godoy P., Veldman K., Duarte F., Acuña M.T., Jarquín C., Weill F.X., Mevius D.J., Wagenaar J.A. (2020). Antimicrobial resistance in *Salmonella enterica* serovar paratyphi B variant Java in poultry from Europe and Latin America. Emerg. Infect. Dis..

[21] Johansson M.H.K., Bortolaia V., Tansirichaiya S., Aarestrup F.M., Roberts A.P., Petersen T.N. (2021). Detection of mobile genetic elements associated with antibiotic resistance in *Salmonella enterica* using a newly developed web tool: MobileElementFinder. J. Antimicrob. Chemother..

[22] Singh T., Dar S.A., Singh S., Shekhar C., Wani S., Akhter N., Bashir N., Haque S., Ahmad A., Das S. (2021). Integron mediated antimicrobial resistance in diarrheagenic *Escherichia coli* in children: In vitro and in silico analysis. Microb. Pathog..

[23] Loaiza K. IntFinder Development and Validation. https://github.com/kalilamali/Integrons.

[24] Zhang A.N., Li L.G., Ma L., Gillings M.R., Tiedje J.M., Zhang T. (2018). Conserved phylogenetic distribution and limited antibiotic resistance of class 1 integrons revealed by assessing the bacterial genome and plasmid collection. Microbiome.

[25] Moura A., Soares M., Pereira C., Leitão N., Henriques I., Correia A. (2009). INTEGRALL: A database and search engine for integrons, integrases and gene cassettes. Bioinformatics.

[26] Cury J., Jové T., Touchon M., Néron B., Rocha E.P. (2016). Identification and analysis of integrons and cassette arrays in bacterial genomes. Nucleic Acids Res..

[27] Hauser E., Tietze E., Helmuth R., Junker E., Prager R., Schroeter A., Rabsch W., Fruth A., Toboldt A., Malorny B. (2012). Clonal dissemination of *Salmonella enterica* serovar Infantis in Germany. Foodborne Pathog. Dis..

[28] Doublet B., Praud K., Nguyen-Ho-Bao T., Argudín M.A., Bertrand S., Butaye P., Cloeckaert A. (2014). Extended-spectrum β-lactamase-and ampc β-lactamase-producing D-tartrate-positive *Salmonella enterica* serovar Paratyphi B from broilers and human patients in Belgium, 2008–2010. J. Antimicrob. Chemother..

[29] Shahada F., Amamoto A., Chuma T., Shirai A., Okamoto K. (2007). Antimicrobial susceptibility phenotypes, resistance determinants and DNA fingerprints of *Salmonella enterica* serotype Typhimurium isolated from bovine in Southern Japan. Int. J. Antimicrob. Agents.

[30] Krauland M.G., Marsh J.W., Paterson D.L., Harrison L.H. (2009). Integron-mediated Multidrug Resistance in a Global Collection of Nontyphoidal *Salmonella enterica* Isolates. Emerg. Infect. Dis..

[31] Marchello C.S., Carr S.D., Crump J.A. (2020). A Systematic Review on Antimicrobial Resistance among *Salmonella* Typhi Worldwide. Am. J. Trop. Med. Hyg..

[32] Cuong N.V., Padungtod P., Thwaites G., Carrique-Mas J.J. (2018). Antimicrobial usage in animal production: A review of the literature with a focus on low-and middle-income countries. Antibiotics.

[33] Fuenmayor Y., Rodas-González A., Carruyo G., Hoet A.E., Wittum T., Narváez-Bravo C. (2019). Salmonella Prevalence and Antimicrobial Drug Resistance in Dual-Purpose Cattle Operations in the Eastern Region of Zulia State, Venezuela. Foodborne Pathog. Dis..

[34] Hartinger S.M., Medina-Pizzali M.L., Salmon-Mulanovich G., Larson A.J., Pinedo-Bardales M., Verastegui H., Riberos M., Mäusezahl D. (2021). Antimicrobial resistance in humans, animals, water and household environs in rural andean peru: Exploring dissemination pathways through the one health lens. Int. J. Environ. Res. Public Health.

[35] World Organisation for Animal Health (OIE) (2020). OEI Annual Report on Antimicrobial Agents Intended for Use in Animals.

[36] Sánchez-Osuna M., Cortés P., Llagostera M., Barbé J., Erill I. (2020). Exploration into the origins and mobilization of di-hydrofolate reductase genes and the emergence of clinical resistance to trimethoprim. Microb. Genomics.

[37] Mejía L., Medina J.L., Bayas R., Salazar C.S., Villavicencio F., Zapata S., Matheu J., Wagenaar J.A., González-Candelas F., Vinueza-Burgos C. (2020). Genomic Epidemiology of *Salmonella* Infantis in Ecuador: From Poultry Farms to Human Infections. Front. Vet. Sci..

[38] Šeputiene V., Povilonis J., Ružauskas M., Pavilonis A., Sužiedeliene E. (2010). Prevalence of trimethoprim resistance genes in Escherichia coli isolates of human and animal origin in Lithuania. J. Med. Microbiol..

[39] Rajaei B., Davar Siadat S., Sepehri Rad N., Badmasti F., Reza Razavi M., Reza Aghasadeghi M., Saboohi R., Rajaei T., Moshiri A., Nejati M. (2014). Molecular Detection of Antimicrobial Resistance Gene Cassettes Associated with Class 2 Integron in *Salmonella serovars* Isolated in Iran. Microbiol. Res. J..

[40] Stern A.L., Van der Verren S.E., Kanchugal S.P., Näsvall J., Gutiérrez-de-Terán H., Selmer M. (2018). Structural mechanism of AadA, a dual-specificity aminoglycoside adenylyl transferase from *Salmonella enterica*. J. Biol. Chem..

[41] Li R., Lai J., Wang Y., Liu S., Li Y., Liu K., Shen J., Wu C. (2013). Prevalence and characterization of *Salmonella* species isolated from pigs, ducks and chickens in Sichuan Province, China. Int. J. Food Microbiol..

[42] Molla B., Miko A., Pries K., Hildebrandt G., Kleer J., Schroeter A., Helmuth R. (2007). Class 1 integrons and resistance gene cassettes among multidrug resistant *Salmonella serovars* isolated from slaughter animals and foods of animal origin in Ethiopia. Acta Trop..

[43] Gassama-Sow A., Diallo M.H., Boye C.S., Garin B., Sire J.M., Sow A.I., Aïdara-Kane A. (2006). Class 2 integron-associated antibiotic resistance in *Shigella sonnei* isolates in Dakar, Senegal. Int. J. Antimicrob. Agents.

[44] Opintan J.A., Newman M.J., Nsiah-Poodoh O.A., Okeke I.N. (2008). Vibrio cholerae O1 from Accra, Ghana carrying a class 2 integron and the SXT element. J. Antimicrob. Chemother..

[45] Li Y., Yang X., Zhang J., Yang S., Zhang S., Chen M., Xue L., Ding Y., Zeng H., Gu Q. (2021). Molecular characterisation of antimicrobial resistance determinants and class 1 integrons of *Salmonella enterica* subsp. enterica serotype Enteritidis strains from retail food in China. Food Control..

[46] Madec J.Y., Doublet B., Ponsin C., Cloeckaert A., Haenni M. (2011). Extended-spectrum β-lactamase blaCTX-M-1 gene carried on an IncI1 plasmid in multidrug-resistant *Salmonella enterica* serovar Typhimurium DT104 in cattle in France. J. Antimicrob. Chemother..

[47] Danel F., Hall L.M.C., Gur D., Livermore D.M. (1997). OXA-15, an Extended-Spectrum Variant of OXA-2-Lactamase, Isolated from a *Pseudomonas aeruginosa* Strain. Antimicrob. Agents Chemother..

[48] Monte D.F.M., Sellera F.P., Lopes R., Keelara S., Landgraf M., Greene S., Fedorka-Cray P.J., Thakur S. (2020). Class 1 integron-borne cassettes harboring blaCARB-2 gene in multidrug-resistant and virulent *Salmonella typhimurium* ST19 strains recovered from clinical human stool samples, United States. PLoS ONE.

[49] Economou V., Gousia P. (2015). Agriculture and food animals as a source of antimicrobial-resistant bacteria. Infect. Drug Resist..

[50] Marshall B.M., Levy S.B. (2011). Food animals and antimicrobials: Impacts on human health. Clin. Microbiol. Rev..

[51] Redding L.E., Cubas-Delgado F., Sammel M.D., Smith G., Galligan D.T., Levy M.Z., Hennessy S. (2014). The use of antibiotics on small dairy farms in rural Peru. Prev. Vet. Med..

[52] Arenas N.E., Abril D.A., Valencia P., Khandige S., Soto C.Y., Moreno-Melo V. (2017). Screening food-borne and zoonotic pathogens associated with livestock practices in the Sumapaz region, Cundinamarca, Colombia. Trop. Anim. Health Prod..

[53] Moser K.A., Zhang L., Spicknall I., Braykov N.P., Levy K., Marrs C.F., Foxman B., Trueba G., Cevallos W., Goldstick J. (2018). The Role of Mobile Genetic Elements in the Spread of Antimicrobial-Resistant Escherichia coli from Chickens to Humans in Small-Scale Production Poultry Operations in Rural Ecuador. Am. J. Epidemiol..

[54] Donado-Godoy P., Clavijo V., León M., Arevalo A., Castellanos R., Bernal J., Tafur M.A., Ovalle M., Alali W.Q., Hume M. (2014). Counts, serovars, and antimicrobial resistance phenotypes of Salmonella on raw chicken meat at retail in Colombia. J. Food Prot..

[55] Abbasoglu D., Akcelık M. (2011). Phenotypic and genetic characterization of multidrug-resistant *Salmonella* Infantis strains isolated from broiler chicken meats in Turkey. Biologia.

[56] Paula Herrera-Sánchez M., Rodríguez-Hernández R., Schroniltgen Rondón-Barragán I. (2020). Molecular characterization of antimicrobial resistance and enterobacterial repetitive intergenic consensus-PCR as a molecular typing tool for *Salmonella* spp. isolated from poultry and humans. Vet. World.

[57] Tanner J.R., Kingsley R.A. (2018). Evolution of Salmonella within Hosts. Trends Microbiol..

[58] Novais Â., Baquero F., Machado E., Cantón R., Peixe L., Coque T.M. (2010). International spread and persistence of TEM-24 is caused by the confluence of highly penetrating Enterobacteriaceae clones and an IncA/C2 plasmid containing Tn1696::Tn1 and IS5075-Tn21. Antimicrob. Agents Chemother..

[59] Franco A., Leekitcharoenphon P., Feltrin F., Alba P., Cordaro G., Iurescia M., Tolli R., D’Incau M., Staffolani M., Di Giannatale E. (2015). Emergence of a Clonal Lineage of Multidrug-Resistant ESBL-Producing *Salmonella* Infantis Transmitted from Broilers and Broiler Meat to Humans in Italy between 2011 and 2014. PLoS ONE.

[60] Bogomazova A.N., Gordeeva V.D., Krylova E.V., Soltynskaya I.V., Davydova E.E., Ivanova O.E., Komarov A.A. (2020). Mega-plasmid found worldwide confers multiple antimicrobial resistance in *Salmonella* Infantis of broiler origin in Russia. Int. J. Food Microbiol..

[61] Silva C., Betancor L., García C., Astocondor L., Hinostroza N., Bisio J., Rivera J., Perezgasga L., Escanda V.P., Yim L. (2017). Characterization of *Salmonella enterica* isolates causing bacteremia in Lima, Peru, using multiple typing methods. PLoS ONE.

[62] Bortolaia V., Kaas R.S., Ruppe E., Roberts M.C., Schwarz S., Cattoir V., Philippon A., Allesoe R.L., Rebelo A.R., Florensa A.F. (2020). ResFinder 4.0 for predictions of phenotypes from genotypes. J. Antimicrob. Chemother..

[63] Clausen P.T.L.C., Aarestrup F.M., Lund O. (2018). Rapid and precise alignment of raw reads against redundant databases with KMA. BMC Bioinform..

[64] Nawrocki E.P., Eddy S.R. (2013). Infernal 1.1: 100-fold faster RNA homology searches. Bioinformatics.

[65] Hall R.M., Brookes D.E., Stokes H.W. (1991). Site-specific insertion of genes into integrons: Role of the 59~base element and determination of the recombination cross-over point. Mol. Microbiol..

[66] Camacho C., Coulouris G., Avagyan V., Ma N., Papadopoulos J., Bealer K., Madden T.L. (2009). BLAST+: Architecture and applications. BMC Bioinform..

[67] Li L.G., Xia Y., Zhang T. (2017). Co-occurrence of antibiotic and metal resistance genes revealed in complete genome collection. ISME J..

